# The Role of Gut Dysbiosis in the Bone–Vascular Axis in Chronic Kidney Disease

**DOI:** 10.3390/toxins12050285

**Published:** 2020-04-29

**Authors:** Pieter Evenepoel, Sander Dejongh, Kristin Verbeke, Bjorn Meijers

**Affiliations:** 1 Laboratory of Nephrology, Department of Immunology and Microbiology, KU Leuven—University of Leuven, B-3000 Leuven, Belgium; 2 Department of Nephrology and Renal Transplantation, University Hospitals Leuven, B-3000 Leuven, Belgium; 3 Translational Research Center for Gastrointestinal Disorders (TARGID), KU Leuven—University of Leuven, B-3000 Leuven, Belgium

**Keywords:** bone, vascular calcification, gut, CKD

## Abstract

Patients with chronic kidney disease (CKD) are at increased risk of bone mineral density loss and vascular calcification. Bone demineralization and vascular mineralization often concur in CKD, similar to what observed in the general population. This contradictory association is commonly referred to as the ‘calcification paradox’ or the bone–vascular axis. Mounting evidence indicates that CKD-associated gut dysbiosis may be involved in the pathogenesis of the bone–vascular axis. A disrupted intestinal barrier function, a metabolic shift from a predominant saccharolytic to a proteolytic fermentation pattern, and a decreased generation of vitamin K may, alone or in concert, drive a vascular and skeletal pathobiology in CKD patients. A better understanding of the role of gut dysbiosis in the bone–vascular axis may open avenues for novel therapeutics, including nutriceuticals.

## 1. Introduction

Chronic kidney disease (CKD) is recognized as a major noncommunicable disease of growing epidemic dimensions worldwide. CDK–mineral and bone disorder (CKD–MBD) is one of the many complications associated with CKD. It represents a systemic disorder of mineral and bone metabolism due to CKD, manifested with either one or a combination of the following: (1) abnormalities of calcium, phosphorus (phosphate), parathyroid hormone, or vitamin D metabolism; (2) abnormalities in bone turnover, mineralization, volume, linear growth, or strength; and (3) vascular or other soft-tissue calcification. CKD–MBD explains, at least in part, the high morbidity and mortality of CKD patients [1].

Bone demineralization and vascular mineralization often concur in CKD, as in the general population. This contradictory association is often referred to as the ‘calcification paradox’ or the bone–vascular axis [2]. Mounting evidence indicates that CKD-associated gut dysbiosis may be involved in the pathogenesis of the bone–vascular axis. The present review aims to update the current evidence on the role of gut dysbiosis in the bone–vascular axis.

## 2. Bone–Vascular Axis

Mounting evidence indicates that CKD is a state of impaired bone quantity [3,4,5,6,7,8,9]. In clinical practice, bone quantity is most commonly assessed by dual-energy X-ray absorptiometry (DXA). A decreased bone quantity [6,10], along with an impaired bone quality [11], contributes to an excessively high fracture risk in CKD patients. Epidemiological evidence demonstrates that the fracture risk increases along with the progression of CKD, with CKD stage-5D patients showing a non-vertebral fracture risk that is up to six times higher than the fracture risk of age- and gender-matched controls [12,13]. Fractures are a major cause of morbidity and, compared to CKD patients without fractures, those with fractures experience a several-fold increased risk of mortality [14,15]. Fractures also impose a large financial burden on healthcare systems. 

Vascular calcification is a condition characterized by calcium phosphate crystal deposition in the intima, media, or cardiac valves [16]. Media calcification is most common among patients with CKD, with prevalence and severity paralleling the progression of renal failure [17]. Vascular calcification is observed in more than 60% of patients with CKD stage 5D [16]. Vascular calcification is an active, cell-regulated process. Its pathophysiology varies across vascular beds and remains incompletely understood, despite major progress in the last decade [18,19,20,21]. Vascular calcification is an established independent risk factor for cardiovascular disease (CVD), the leading cause of morbidity and mortality in patients with CKD [22,23]. 

Many clinical studies have demonstrated an association between low bone mass and vascular calcification in patients with CKD [24,25,26,27,28,29]. The association between osteoporosis and vascular calcification is not specific to CKD. It also is commonly observed in the elderly and in patients with diabetes mellitus or chronic obstructive pulmonary disease [30,31,32,33,34,35]. Importantly, the association remains significant after adjustment for age, which suggests an age-independent relationship [26,27,29,30,31,32,33,36,37]. Vascular calcification and bone mineralization are both actively regulated processes showing many similarities. The co-existence of bone loss with vascular calcification should therefore be considered a paradoxical phenomenon. It is commonly referred to as the ‘calcification paradox’. It most likely reflects direct bone–vascular cross-talk and/or the involvement of common pathogenic factors [2,35]. 

## 3. Gut Microbial Ecosystem in Health and CKD

The human gut harbors a complex and dynamic microbial ecosystem that is shaped by diet and host factors [38]. The human microbiome project has shown that the composition of the microbial ecosystem is quite different from one individual to the other. This variability in composition is not continuous and random, but stratified. Nutrient intake patterns are associated with both the degree of diversity and certain clusters of microbial species that are often found to act in concert. The microbial ecosystem thrives on the nutritional leftovers brought to them via the digestive tract. This requires substantial metabolic flexibility, as nutrient availability is dependent on host nutrient intake and digestion. A complex web of overlapping metabolic pathways allows access to nutritional sources inaccessible to mammalian metabolism, thereby supplementing the host metabolism. 

The gut microbiota provides the host with a variety of functions including the digestion of complex dietary components, production of vitamins, maturation of the immune system, protection against pathogens, and regulation of host metabolism [39]. A compelling set of bidirectional links between the gut microbiota and the host (patho) physiology has emerged, and metabolites produced by the microbiota are increasingly implicated as crucial executors of the microbial influence on the host. Of note, microbial metabolites do account for about 10% of circulating metabolites [40]. 

CKD is associated with a disturbed gut microbiota composition and metabolism [41,42,43]. These disturbances reflect the aggregate consequences of CKD, more specifically, the effects of kidney dysfunction combined with the effects of therapeutic interventions and dietary modifications. Kidney dysfunction has a major impact on a number of physiological systems, including the gastrointestinal tract. More specifically, gastrointestinal assimilation and motility, both known to modify the colonic microenvironment, may be disturbed in CKD [44,45]. CKD, furthermore, causes an increased influx of urea, uric acid, and oxalate into the colon. Urea is converted to ammonia and subsequently to ammonium hydroxide, which can raise the colonic pH and result in mucosal damage. Patients with CKD, furthermore, often consume a diet low in dietary fiber to avoid hyperkaliemia. These and other dietary measures may importantly impact on gut microbiota composition and metabolism. Finally, not only antibiotics, but also non-antibiotic drugs are increasingly recognized to extensively affect human gut bacteria [46]. This is especially relevant in the setting of CKD, as pill burden in these patients is huge. 

Using bacterial DNA isolated from fecal samples, Vaziri et al. showed highly significant differences in the abundance of over 200 bacterial operational taxonomic units between hemodialysis patients and healthy controls [41]. Additional studies demonstrated that patients with End Stage Kidney Disease (ESKD) had an increased number of bacteria that possess urease, uricase, and p-cresol- and indole-forming enzymes, and a contraction of families or genera possessing butyrate-forming enzymes (e.g., *Roseburiae*, *Lactobacillaceae*, and *Prevotellaceae*) [47,48]. Metabolomics studies showed clear differences in the levels of fecal metabolites (including phenols, indoles, and aldehydes) between patients with CKD and healthy controls. Of interest, the differences in fecal metabolite profiles were greater between patients on hemodialysis and unrelated healthy individuals than between patients on hemodialysis and household members exposed to the same diet [43]. Gryp et al., conversely, failed to observe increasing levels of p-cresyl sulfate, p-cresyl glucuronide, indoxyl sulfate, indole-3-acetic acid levels, and their precursors in stool and urine samples of patients along with the progression of CKD. In addition, anaerobic culture of fecal samples showed no difference in ex vivo p-cresol, indole, and indole-3-acetic acid generation (https://doi.org/10.1016/j.kint.2020.01.028). The use of animal models enables the effects of CKD to be separated from those of therapeutic interventions and diet. Studies with uremic rats confirm that renal dysfunction itself induces profound changes in the gut microbiota composition [41] and metabolism [43]. Taken together, current evidence indicates that CKD causes a microbial metabolism shift away from saccharolytic fermentation and towards proteolytic fermentation. Given some contradictory findings, additional prospective studies are required to confirm this shift.

CKD-induced changes to the composition and function of the intestinal microbiota also impair the intestinal barrier function, a condition commonly referred to as leaky gut [38]. A leaky gut in CKD is evidenced by the observation of increased concentrations of bacterial components, such as endotoxin or DNA, in the circulation of patients with increasing CKD stage. The levels of bacterial components are the highest in patients with ESKD treated with dialysis [49,50]. Although circulating bacterial components in patients on dialysis might derive from external sources such as dialysate fluids, the intestinal microbiota is by far the most likely source of these components in patients with CKD not on dialysis [50]. One study showed that after a few days of feeding uremic rodents with a non-pathogenic but green fluorescent *Escherichia coli* strain, green fluorescent bacterial colonies could be cultured from mouse livers, demonstrating that CKD facilitates the translocation across the intestinal barrier not only of bacterial components but also of entire living bacteria [51,52]. Our current understanding of the effects of CKD on the intestinal barrier function is in line with studies from the 1990s that demonstrated that orally ingested high-molecular-mass polyethylene glycols cross the intestinal barrier and enter the circulation and urine of uremic animals and patients [53]. Some but not all studies in animal models of CKD have demonstrated superficial mucosal erosions or disrupted tight junctions between intestinal epithelial cells in several parts of the gastrointestinal tract [52,54,55], in line with autopsy findings of patients on maintenance hemodialysis, which frequently show subtle pathologies indicative of diffuse gastrointestinal wall inflammation. Both an increased exposure to urea-derived ammonia and ammonium hydroxide [56] and a decreased generation of butyrate may contribute to a leaky gut [57]. Butyrate maintains the barrier function by at least two not mutually exclusive mechanisms. Butyrate is the primary energy source for colonic epithelial cells and undergoes fatty-acid oxidation to such an extent that these cells are slightly hypoxic. This leads to hypoxia-inducible factor-1-mediated upregulation of tight junction genes [58]. In addition, butyrate functions as a histone deacetylase (HDAC) inhibitor, and this has been shown to upregulate tight junction genes as well as the major intestinal mucin *MUC2* [59,60] gene and to downregulate the expression of pro-inflammatory cytokines [61]. Treatment of uremic rats with the symbiont *Bifidobacterium animalis* subsp. lactis Bi-07 attenuated epithelial erosion and decreased intestinal inflammation [52]. 

## 4. Gut–Bone–Vascular Axis in CKD

**Figure 1:**
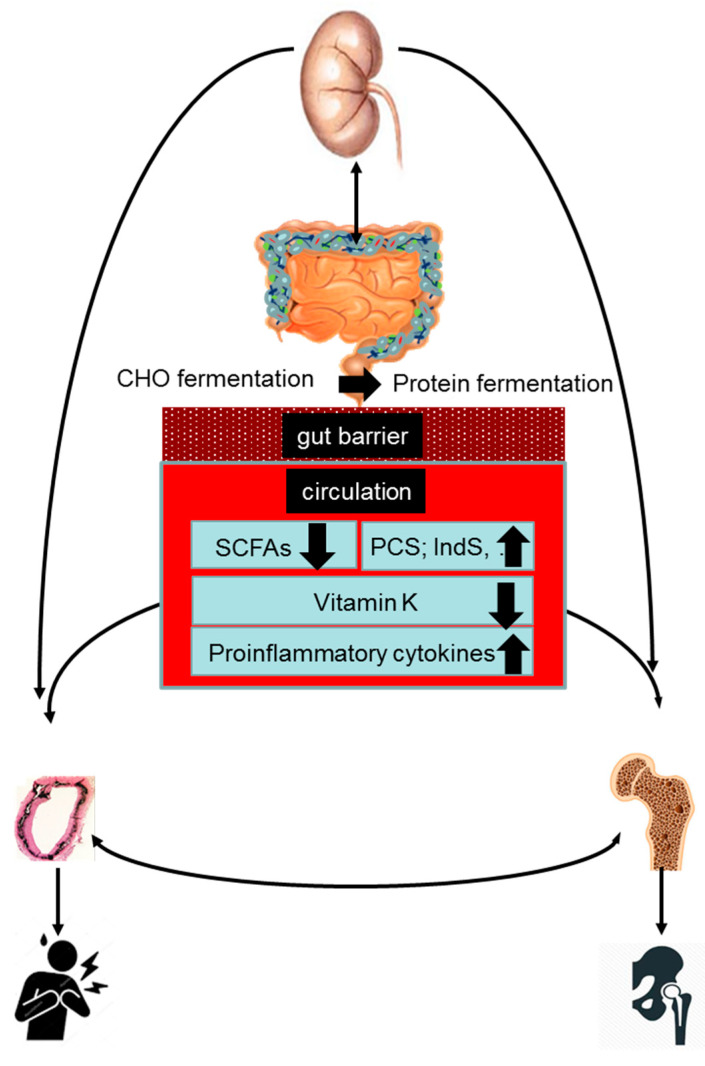
The kidney–gut–bone–vascular axis. Chronic kidney disease is associated with gut dysbiosis, characterized by a metabolic shift towards a predominantly proteolytic fermentation pattern and a leaky gut. Gut dysbiosis may induce bone loss and vascular calcification and as such may play a pathogenic role in the bone–vascular axis in CKD. Underlying pathophysiological mechanisms include increased exposure to protein fermentation metabolites (such as p-cresyl sulfate (PCS) and indoxyl sulfate (IndS)), a leaky gut contributing to inflammation, and deficiency of vitamin K and short-chain fatty acids (SCFAs).

Acknowledging that the gut microbiome is a key regulator of bone [62,63,64] and cardiovascular [65,66,67] health, gut dysbiosis may be hypothesized to be involved in the pathogenesis of the bone–vascular axis. The present review discusses mechanisms by which gut dysbiosis may contribute to vascular calcification and bone demineralization in the setting of CKD. We herein will separately discuss the role of increased protein fermentation, decreased carbohydrate fermentation, vitamin K deficiency, and gut-derived inflammation (Figure 1). 

## 5. Role of Increased Protein Fermentation in the Bone–Vascular Axis

End products of protein fermentation such as phenols and indoles are largely [68] transported across the colonic epithelium via active and passive transport mechanisms [57,69] and subsequently metabolized by phase 1 and 2 reactions (e.g., towards *p*-cresyl sulfate (PCS) and indoxyl sulfate (IndS)) in the colonic epithelium and liver before entering the systemic circulation 70. Whether CKD affects transport kinetics and metabolism of protein fermentation metabolites remains to be investigated. Protein fermentation metabolites are cleared from the circulation by the kidneys, mainly by tubular secretion, since most are strongly protein-bound [70]. Plasma concentrations of PCS and IndS increase along the progression of CKD to reach levels in patients with ESKD being 10- to 50-fold higher than in healthy controls. These high levels reflect both an increased intestinal production and absorption and a decreased renal clearance [71]. At uremic concentrations, PCS and IndS may disturb several biological processes and confer direct and indirect toxicity in various cells and tissues, at least partly by generating intracellular oxidative stress [72]. 

Experimental studies revealed that IndS and PCS may promote vascular calcification through various mechanisms [73,74,75]. These mechanisms include (a) increased shedding of endothelial microparticles [76,77], (b) impaired autophagic flux in endothelial cells [78], (c) downregulation of MiR-29b [79], and (d) suppression of the nuclear factor erythroid 2-related factor 2 (NRF2), a master regulator of cellular antioxidant activity [80]. Dahl salt-sensitive hypertensive IndS-administered rats presented aortic calcification and upregulation of osteogenic genes when compared to control rats, indicating a pro-calcifying role of IndS in an in vivo animal model [81]. In a subsequent experiment by the same group, Dahl salt-sensitive hypertensive IndS-administered rats presented markers of senescence in the area of aortic calcification [82]. Recently, Opdebeeck et al. reported that both IndS and PCS independently promote vascular calcification in the adenine-induced CKD rat model. This was demonstrated in the aorta, as well as in peripheral arteries. Uremic toxin-induced vascular calcification was associated with the activation of inflammation and coagulation pathways [83]. 

In line with these experimental data, the circulating levels of PCS and IndS have been repeatedly associated with cardiovascular morbidity (including arterial stiffness, vascular calcification, ischemic and thrombotic events, and atrial fibrillation) and mortality in patients with CKD across stages of the disease [84,85,86] Also in the general population, clear associations between PCS and IndS concentrations and cardiovascular endpoints have been reported. For example, in a population-based study in Belgium, the prevalence of hypertension increased along with PCS and IndS quartiles [87].

Evidence of the skeletal toxicity of protein fermentation metabolites is much more limited. Protein fermentation metabolites may confer direct toxicity to bone cells and disrupt bone matrix characteristics, thereby compromising bone quality and strength [88,89]. IndS promotes osteoblast apoptosis [90] and inhibits osteoclast differentiation [91]. The latter may occur through aryl hydrocarbon receptor signaling-dependent suppression of receptor activator of nuclear factor kappa-Β ligand (RANKL) production [92]. IndS also causes the deterioration of bone mechanical properties [93,94] and bone architecture. Finally, IndS may induce skeletal resistance to parathyroid hormone (PTH) [95]. Increased protein fermentation may contribute to the high prevalence of a dynamic bone disease in patients with CKD, despite these patients often presenting with PTH levels exceeding the normal upper limit severalfold [96].

Protein fermentation metabolites may also affect bone and vascular health indirectly, e.g., by promoting inflammation (vide infra) and epigenetic silencing of Klotho, an anti-aging protein [97,98,99]. Emerging evidence indicate that Klotho deficiency is involved in the pathogenesis of vascular calcification and bone loss in CKD. *Klotho*-null mice [100,101] show extensive vascular calcification and a low-turnover osteopenia phenotype. The bone phenotype, most probably, results from systemic disturbances in mineral metabolism associated with disrupted FGF23–Klotho signaling rather than from a functional defect of Klotho in osteocytes [102,103].

## 6. Role of Decreased Carbohydrate Fermentation in the Bone–Vascular Axis

Fermentation of complex carbohydrates results in the generation of short-chain fatty acids (SCFAs) [104]. The main SCFAs are butyrate, propionate, and acetate, which are found in the intestine in a molar ratio of 60:20:20. SCFAs are efficiently absorbed by the gut mucosa by poorly selective anion-transporting proteins [105]. SCFAs, not used by the colonocytes as a source of energy, enter the portal circulation and subsequently either are metabolized by the liver or enter the systemic circulation. SCFAs entering the systemic circulation have important impacts on host physiology as sources of energy, regulators of gene expression (e.g., via inhibition of HDAC), and signaling molecules that are recognized by specific receptors. Especially butyrate is a pleiotropic molecule, functioning as a ligand for certain G protein-coupled receptor (GPCR, e.g., GPCR41 and 43, also known as free-fatty acid receptor 3 and 2) and as a peroxisome proliferator-activated receptor agonist [57]. 

Production of both propionate and butyrate is reduced in animal CKD models [106]. Human studies, so far, yielded inconsistent findings with regard to both the overall capacity of microbiota to produce butyrate [107] and the circulating levels of SCFAs [108,109]. Chinese patients with CKD stage 5 showed a reduction in the most abundant butyrate-producing microbial species 48 and almost threefold lower plasma butyrate levels than healthy controls [108]. A comparable study in the Netherlands, however, failed to confirm these findings [107]. 

An increasing body of evidence implicates SCFAs in the pathogenesis of bone disease [64]. SCFAs may promote a positive bone balance by suppressing osteoclastogenesis and stimulating osteoblastogenesis. Mechanistically, propionate and butyrate induce metabolic reprogramming of osteoclasts, resulting in enhanced glycolysis at the expense of oxidative phosphorylation, thereby downregulating essential osteoclast genes [110]. Butyrate, furthermore, suppresses osteoclast differentiation, most probably by increasing the production of osteoprotegerin (OPG) by human osteoblasts [111,112]. Butyrate is also capable of stimulating bone formation [111,113]. The underlying mechanisms remain poorly defined. Butyrate promotes the differentiation of naïve CD4^+^ cells into regulatory T cells (Tregs). The expansion of Tregs in the bone marrow leads to increased production of Wnt10b. This Wnt ligand subsequently activates Wnt signaling in osteoblastic cells, leading to osteoblast proliferation, differentiation, and survival [113]. Remarkably, this anabolic effect is only seen in trabecular bone. It is unclear whether, and if so, to what extent, the weak inhibition of HDACs accounts for the bone anabolic effects of butyrate [114].

SCFAs may also protect bone indirectly, e.g., by suppressing inflammation (vide infra) and by increasing insulin-like growth factor 1 (IGF-1), a distinct bone anabolic factor [115]. Finally, the CKD-induced microbial metabolism shift away from saccharolytic fermentation and towards proteolytic fermentation creates a colonic microenvironment (e.g., a higher luminal pH) that may hamper calcium absorption [62]. The contribution of calcium absorption in the colon to the overall calcium influx is probably limited. Nevertheless impaired colonic calcium absorption may contribute to a tight, if not negative, calcium balance, commonly observed in CKD patients free of calcium supplements [116]. 

Studies exploring the role of SCFAs in vascular (patho)biology are limited. Butyrate activates NRF2 at the transcription level [117,118,119,120]. This effect is mediated by HDAC inhibition. One of the downstream effects of NRF2 activation is the upregulation of the glutathione/glutathione S-transferase (GST) antioxidant system resulting in a beneficial smooth muscle cell (VSMC) redox state [121]. Activation of NRF2 signaling has been shown to alleviate high phosphate-induced calcification of VSMCs [122]. SCFAs also have anti-inflammatory properties and thus may indirectly protect against vascular calcification (vide infra).

## 7. Role of Vitamin K Deficiency in the Bone–Vascular Axis

Microbiota are capable of producing menaquinones (vitamin K2). To what extent the microbial production of menaquinones (vitamin K2) contributes to the overall vitamin K status of the host remains a matter of ongoing debate [123]. Experimental studies on the effect of oral and colorectal administration of vitamin K on circulating prothrombin concentration in vitamin K-deficient rats demonstrated that the bioavailability of colonic vitamin K is more than 50-fold lower than the bioavailability of oral vitamin K [123]. Conversely, data from germ-free rodents [124] and experimental and clinical studies with broad-spectrum antibiotics indicate that gut microbial metabolism may be important to maintain adequate vitamin K stores in the mammalian host [125,126,127].

Recent data indicate that a large majority of patients with CKD are vitamin K-deficient [128,129,130,131,132,133]. Besides dietary restrictions, therapy with vitamin K antagonists and phosphate chelators, and impaired vitamin K recycling, a decreased microbial production related to gut dysbiosis may account for the high prevalence of functional vitamin K deficiency in CKD [129,130,134,135]. 

Vitamin K deficiency is a well-recognized risk factor of vascular calcification and arterial stiffness, both in the general population and in CKD patients [136,137]. Accelerated vascular calcification in individuals with functional vitamin K deficiency is explained by incomplete γ-carboxylation and reduced function of matrix Gla protein (MGP) in the vasculature [138]. MGP is a 14 kDa secretory protein synthesized by chondrocytes, VSMCs, endothelial cells (ECs), and fibroblasts. γ-Carboxylated MGP inhibits vascular mineralization both directly, as a part of a complex with fetuin-A (also known as α-2-HS-glycoprotein), and indirectly, by interfering with the binding of bone morphogenetic protein-2 (BMP-2) to its receptor and thereby inhibiting BMP-2-induced osteogenic differentiation. 

Low dietary intake of vitamin K, therapy with vitamin K antagonists, and functional vitamin K deficiency, as determined by circulating biomarkers (such as dephosphorylated–uncarboxylated MGP), are associated with low bone mineral density (BMD) and increased risk of fractures, both in the general population [90,91,92] and in patients with CKD [133,139]. Vitamin K-dependent γ-carboxylation of Gla-containing bone proteins such as MGP and osteocalcin (also referred to as bone Gla protein) may positively impact mineralization and bone quality. However, much remains to be learned on the role of MGP and osteocalcin in bone biology [140,141,142]. Vitamin K may affect bone health also directly by targeting the steroid and xenobiotic-sensing nuclear receptor (SXR), expressed in osteoblasts [141,143]. Finally, vitamin K deficiency may trigger micro-inflammation and thus contribute to the calcification paradox (vide infra) [133,144]. 

## 8. Role of Inflammation in the Bone–Vascular Axis

CKD is well-recognized as a state of micro-inflammation [25,145]. Several factors contribute to the inflammatory status in CKD. Only in recent years, gut dysbiosis has been recognized as another important culprit [54,146]. The pathways linking gut dysbiosis to inflammation are manifold. 

First, gut dysbiosis is associated with a dysfunctional epithelial barrier [69,147]. The disruption of the gut epithelial barrier enables the entry of endotoxin and other microbial components into the systemic circulation, which in turn may elicit an inflammatory response [148]. Several studies in animal models of CKD have documented superficial mucosal erosions, mucin loss, or disrupted tight junctions between intestinal epithelial cells in several parts of the gastrointestinal tract [54,55,149,150], in line with autopsy findings in patients on chronic hemodialysis, which who show subtle pathologies indicative of diffuse gastrointestinal wall inflammation [151]. Besides gut dysbiosis, sympathetic overactivity and intestinal congestion due to hypervolemia as a result of heart failure are hypothesized to contribute to increased intestinal permeability in CKD [38]. 

Second, both an increased exposure to protein fermentation metabolites and a decreased exposure to SCFAs have been hypothesized to contribute to micro-inflammation in CKD. PCS was shown to activate leucocyte free-radical production [152] and IndS-induced proinflammatory cytokines in human primary macrophages, by a mechanism involving the activation of Delta-like 4 (Dll4)–Notch signaling [153]. Other studies, conversely, failed to confirm the proinflammatory properties of PCS and IndS [154]. Moreover, clinical studies investigating the relationship between serum levels of gut-derived uremic toxins, markers of inflammation, yielded inconsistent findings [155]. The anti-inflammatory immune-regulatory properties of circulating SCFAs are well established and best characterized for butyrate. Butyrate stimulates the production of ketone bodies, including β-hydroxybutyrate, known to suppress the activation of the NACHT leucine-rich repeat and pyd domains-containing 3 (NLRP3) inflammasome [156] and suppresses nuclear factor kappa-Β (NF- kappa-Β) signaling in immune cells [157,158]. Butyrate may also mediate systemic anti-inflammatory effects by inhibition of HDACs [57,159]. However, the clinical relevance of the latter mechanism is questionable, as butyrate circulates only at micromolar levels, which is far below the IC50 for HDAC inhibition. 

Finally, vitamin K deficiency is associated with inflammation [133,144]. Both causality of the relationship and its underlying molecular mechanisms remain to be defined. 

Inflammation may be a common soil for bone loss and vascular calcification [24,25,160,161,162,163,164,165,166]. The pathophysiological mechanisms linking inflammation to vascular calcification are complex and multifaceted. Inflammatory cytokines and C-reactive protein may (a) promote endothelial-to-mesenchymal transition [161], (b) augment osteo–chondrogenic differentiation of vascular smooth muscle cells through activation of Msx2–Wnt/β-catenin signaling [167] and induction of oxidative stress [168], and (c) repress the production of fetuin-A, an important calcification inhibitor [169]. Vascular calcification, in turn, may elicit an inflammatory response and as such trigger a self-perpetuating vicious circle.

Experimental data indicate that inflammatory cytokines, either circulating or locally produced in the bone, such as TNF-α, IL-6, and IL-1β, may induce increased bone resorption [170,171,172,173]. These effects are mediated, in part, via cytokine-induced increases in RANKL, a key stimulator of bone resorption, expressed by osteoblasts and T cells [174]. TNF-α is also an inhibitor of bone formation [175], further tilting the balance towards bone loss [161]. In disagreement with these data, Barreto et al., reported a positive correlation between TNF-α levels and bone area [176]. These authors speculate that elevated TNF-α expression may represent a homeostatic feedback mechanism to counteract excessive bone mass gain. 

## 9. Therapeutic Options

Targeting gut microbiota composition and metabolism may be appealing to tackle the immense burden of cardiovascular disease and fractures simultaneously. Human trials and experimental murine models have shown that nutrition (e.g., diets high in dietary fiber), probiotics, prebiotics, or supplementation of SCFAs may have beneficial effects on the skeleton [64,177,178] and cardiovascular system [179]. The potential of food as a medicine in the setting of CKD is huge, but many questions remain with regard to the optimal use of nutriceuticals. A ‘one-size-fits-all’ approach is unlikely to be successful. Omics, undoubtedly, will prove useful in personalizing nutritional therapy [180,181].
